# Varied Manifestations of Sharp Penetrating Foreign Bodies in the Aerodigestive Tract: Our Experience

**DOI:** 10.7759/cureus.39525

**Published:** 2023-05-26

**Authors:** Nisha V, K C Prasad, Induvarsha G, Kouser Mohammadi

**Affiliations:** 1 Otolaryngology, Sri Devaraj Urs Academy of Higher Education and Research, Kolar, IND; 2 Otolaryngology/Head and Neck Surgery, Sri Devaraj Urs Academy of Higher Education and Research, Kolar, IND

**Keywords:** aerodigestive tract, sharp penetrating foreign bodies, foreign body, sharp penetrating, ingestion, aspiration

## Abstract

Introduction

Foreign body ingestion or aspiration is an emergency dealt by otorhinolaryngologists. It is most common among children and the geriatric population. It paves the way for critical morbidity when prompt treatment is not initiated. Therefore, in the absence of strong evidence to guide decision-making, all suspicious presentations of the ingested sharp foreign body need to be kept in mind while making a diagnosis. Hence, our study is aimed to document the varied manifestations of sharp penetrating foreign bodies in the aerodigestive tract.

Materials and methods

The medical records of 40 patients who presented with sharp foreign body ingestion/aspiration in the department of otorhinolaryngology in our centre from September 2012 to September 2022 were reviewed retrospectively.

Results

In all 40 patients, we were able to retrieve the foreign body as such without crushing or breaking it. In our study, the most common foreign body retrieved among middle-aged and elderly were chicken bone (22.5%) or fish bone (25%), and the most common foreign body following accidental ingestion in children were stapler pins (20%).

Conclusion

The findings of our study concluded that relevant clinical history, atypical presentation, and radiological imaging of sharp penetrating foreign bodies in the neck should be addressed with the utmost caution, as foreign bodies migrate to deep neck space and bronchus and can result in untoward complications. Hence, we need to be suspicious of the varied manifestation of aerodigestive tract foreign bodies for early diagnosis and prompt treatment.

## Introduction

Foreign body ingestion or aspiration is an emergency dealt by otorhinolaryngologists. It is common among children and the geriatric population. It paves the way for critical morbidity when prompt treatment is not initiated [1]. Despite the upgradation of people's awareness and emergency services, oesophageal foreign bodies in children still prevail, causing 1500 deaths per year [2]. Commonly encountered foreign bodies include bones, pieces of glass, coins, and needles. The most common sites for foreign body penetration are the hypopharynx and cervical oesophagus [3,4].

Various complications due to foreign body ingestion include retropharyngeal abscess, tracheoesophageal fistula, perforation, aspiration pneumonia, and mediastinitis, and can lead to septicaemia, acute bleeding, and shock, necessitating the need for immediate management. The movement of foreign bodies in the aerodigestive tract attributes a considerable share of varied complications and modes of management. Radiological investigations help in understanding the type of foreign body, its extent, and its relation to vital structures, thereby aiding in early diagnosis. In the modern era, CT scan has become the diagnostic method of choice over X-rays, with its sensitivity and specificity being 90% and 100%, respectively [3,4].

Recently, there are various treatment options available for foreign body removal. The mode of management changes based on its varied manifestations in the aerodigestive tract. Endoscopic removal of the foreign body is routinely practised, but it does not always hold well in the case of a sharp foreign body. Various techniques and endoscopic equipment are used to remove sharp ingested foreign bodies in children, including specialized forceps, snares, and friction-fit adapters.

Although early impaction of the foreign body in the upper aerodigestive tract has been reported, there is limited knowledge regarding its varied presentations. Hence early diagnosis and prompt treatment become a challenge, especially in those cases presenting with atypical manifestations. The literature also has a low level of evidence. Therefore, in the absence of strong evidence to guide decision-making, all suspicious presentations of ingested sharp foreign bodies need to be kept in mind while making a diagnosis. Hence, our study is aimed to document the varied manifestations of sharp penetrating foreign bodies in the aerodigestive tract.

## Materials and methods

In this retrospective observational study, varied manifestations of sharp penetrating foreign bodies in the aerodigestive tract were reviewed. An Institutional Ethics Committee clearance was taken prior to the start of the study from Sri Devaraj Urs Academy of Higher Education and Research (DMC/KRL/IEC/607/2022-2023). Medical records of all patients presenting with sharp foreign body ingestion/aspiration in the department of otolaryngology of our tertiary rural health centre from September 2012 to September 2022 were traced retrospectively. Written informed consent was taken from all the participants to access their clinical data. The presenting symptoms, past history, type of foreign body, and radiological investigations were reviewed. All patients underwent emergency exploratory surgery. Data were collected retrospectively from the case records of the patients. Data analysis was done by using SPSS software version 24 (IBM Corp., Armonk, NY). Qualitative data were described as frequency and percentages.

## Results

Out of 40 patients, 25 were male and 15 were female. The majority of them were of paediatric age, as shown in Table 1.

**Table 1:** Age and gender-wise distribution of study participants (n = 40)

Age group	Male	Female
<10	7 (17.5)	12 (30)
11-20	0 (0)	1 (2.5)
21-30	3 (7.5)	1 (2.5)
31-40	3 (7.5)	0 (0)
41-50	4 (10)	1 (2.5)
>50	8 (20)	0

All 39 patients had a history of foreign body ingestion, and one patient had a history of injury with a high-velocity object in the anterior aspect of the neck.

The most common presentations among 40 patients were a pricking sensation in the throat, pain, and difficulty in swallowing.

All patients presented within 72 hours of chief complaints. All of them underwent X-rays of the neck and chest. In addition, computed tomography with or without contrast was performed in selected stable patients along with routine blood investigations. In the majority of patients (35%), foreign body removal was done via rigid oesophagoscopy, followed by 25% who underwent direct laryngoscopy, 20% underwent rigid bronchoscopy, 15% underwent incision and drainage and foreign body removal (via extraoral/intraoral route), 2.5% underwent neck exploration and rigid bronchoscopy, and 2.5% underwent thoracotomy, as shown in Table 2.

**Table 2:** Different procedures adopted for foreign body removal

Type of procedure	Number of cases (n)	Frequency (%)
Direct laryngoscopy	10	25%
Rigid oesophagoscopy	14	35%
Rigid bronchoscopy	08	20%
Incision and drainage (intra-oral approach)	04	10%
Incision and drainage (extra-oral approach)	02	05%
Neck exploration + rigid bronchoscopy	01	2.5%
Thoracotomy	01	2.5%

Sites of penetration of the foreign body were also assessed intraoperatively, and the most common sites were the cricopharynx (27.5%), bronchus (22.5), and vallecula (17.5%), respectively, as shown in Table 3.

**Table 3:** Various sites of penetration of the foreign body

Site of penetration	No. of patients	%
Tonsillar fossa	1	2.5
Parapharyngeal space	2	5
Post-pharyngeal wall	1	2.5
Retropharyngeal space	1	2.5
Median glossoepiglottic fold	1	2.5
Vallecula	7	17.5
Pyriform fossa	1	2.5
Postcricoid	4	10
Cricopharynx	11	27.5
Oesophagus	2	5
Bronchus	9	22.5

In all 40 patients, we succeeded in removing the foreign body as such without crushing or breaking it. In our study, the most common foreign body retrieved among middle-aged and elderly were chicken bone (22.5%) or fish bone (25%), and the most common foreign body following accidental ingestion in children were stapler pins (20%), as shown in Table 4.

**Table 4:** Various foreign bodies retrieved

Various foreign bodies	No. of patients	%
Stapler pin	8	20
Denture	4	10
Fish bone	10	25
Chicken bone	9	22.5
Stone	3	7.5
Metal piece	4	10
Safety pin	2	5

Out of 40 patients, all 30 patients had similar presentations, and 10 patients had varied manifestations, as shown in Table 5.

**Table 5:** Varied presentations of aerodigestive tract foreign bodies

Presentations	No. of patients	%
Laceration in the anterior aspect of the neck, thyroid ala cut	1	2.5
Parapharyngeal abscess	2	5
Migration to retropharyngeal space	1	2.5
Retropharyngeal abscess	2	5
Aspiration pneumonia	3	7.5
Oesophagitis	1	2.5
Other common manifestation	30	75

Case details and presentation of 10 patients documented in detail

A middle-aged man who was a blacksmith by occupation presented with a history of injury to the anterior aspect of the neck by a high-velocity metallic object encountered at his workplace. This was followed by bleeding from the site of injury, haemoptysis, and a change in voice. There was a 6-mm laceration just below the thyroid notch. X-ray of the neck and chest revealed a radio-opaque foreign body in the right main bronchus. Rigid bronchoscopy and neck exploration were done for the same, and a metallic foreign body with sharp edges (missile) measuring 1.5 x.7 cm was seen near the opening of the middle lobe in the medial segment bronchus of the right main bronchus, along with hematoma of the right ventricular fold. The encountered foreign body was not obstructing the airway and there were no signs of bleeding or ulceration of the bronchial mucosa. The foreign body was removed with no impending complications. The neck wound was closed in layers, and the hoarseness of voice improved postoperatively.

An elderly person presented with cheek swelling, fever, throat pain with blood-tinged vomitus with a history of chicken bone ingestion three days ago. He was a known diabetic on irregular medication. On examination, there was diffuse swelling in the right parotid region, the right tonsil was pushed medially, erythematous faucial pillars were noted, and palpable jugulodigastric lymph nodes were present on the same side. X-ray of the neck and chest showed soft tissue thickening in pre-vertebral space. Contrast-enhanced computed tomography (CECT) of the neck was done, which showed a right parapharyngeal abscess. The patient received prophylactic antibiotics and later underwent emergency tracheostomy with incision and drainage via extraoral approach, followed by removal of chicken bone impacted in the right tonsillar fossa. Postoperatively, the patient started receiving antibiotic coverage. The feeding tube was inserted and decannulated after two days.

Another middle-aged patient presented with fever, pain, and difficulty in swallowing, with a suspected history of chicken bone ingestion three days back. On examination, there were erythematous faucial pillars and bulges in the left lateral aspect of the posterior pharyngeal wall. X-ray of the neck showed a widening of pre-vertebral space. CT of the neck was taken, which showed a left parapharyngeal abscess. Incision and drainage were done via the intra-oral route along with tracheostomy, but no foreign bone was found. A repeat CT of the neck showed the absence of any foreign body, which hinted that the foreign body present in the parapharyngeal space would have been suctioned out during the time of incision and drainage.

**Figure 1:**
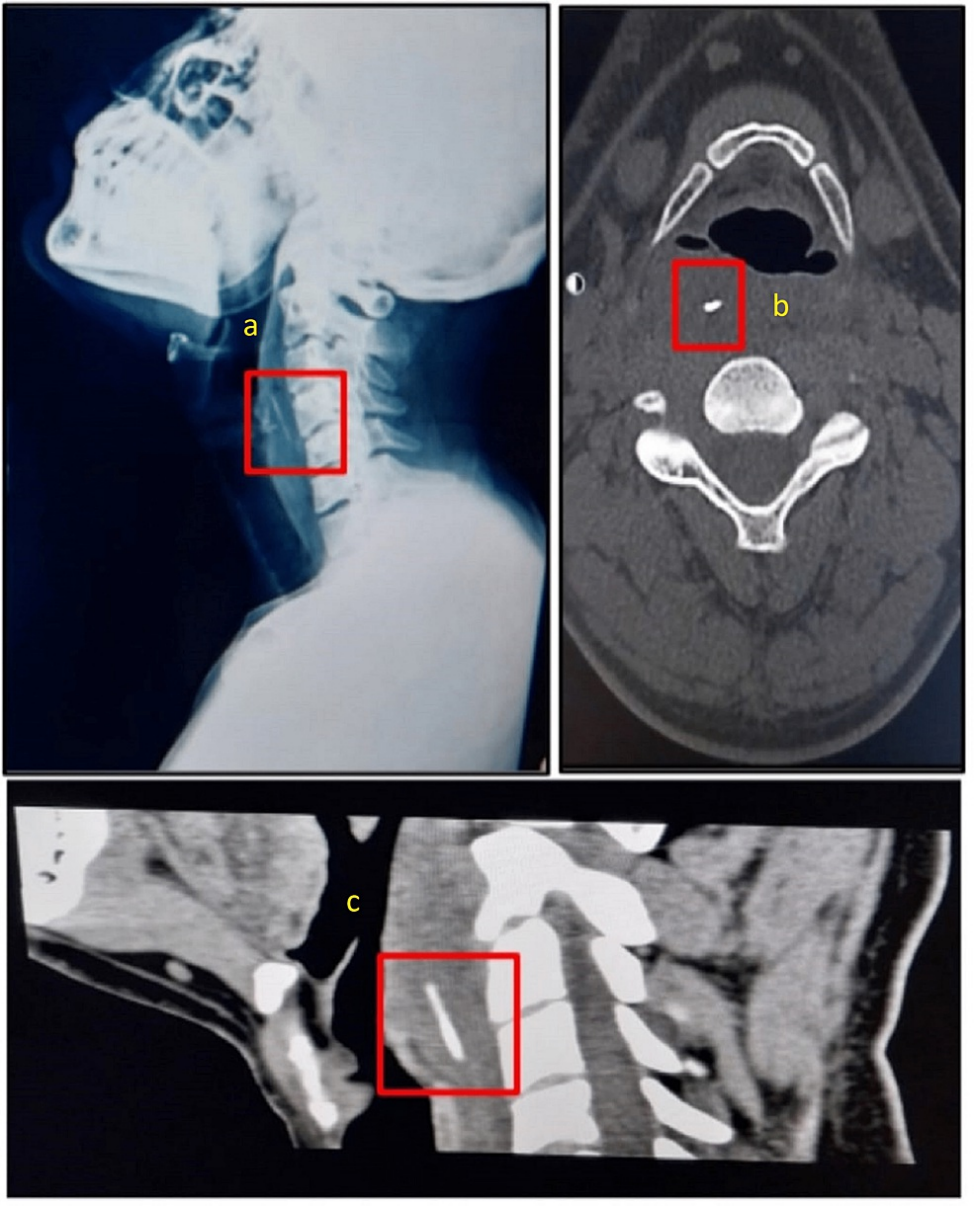
Radiological investigations suggestive of a foreign body (a) Plain X-ray of the head and neck (lateral view) showing a radio-opaque foreign body at the level of the hyoid bone. (b) Axial CT image showing the linear hyperdense structure at the level of the hyoid bone, likely a foreign body. (c) Sagittal reformatted plain CT showing a 2.1 cm linear hyperdense structure extending obliquely and posteromedially from hyoid to apex of pyriform fossa, 1 cm lateral to the midline, and 1 cm in depth from the posterior pharyngeal wall at C2 vertebral level, likely a foreign body.

**Figure 2:**
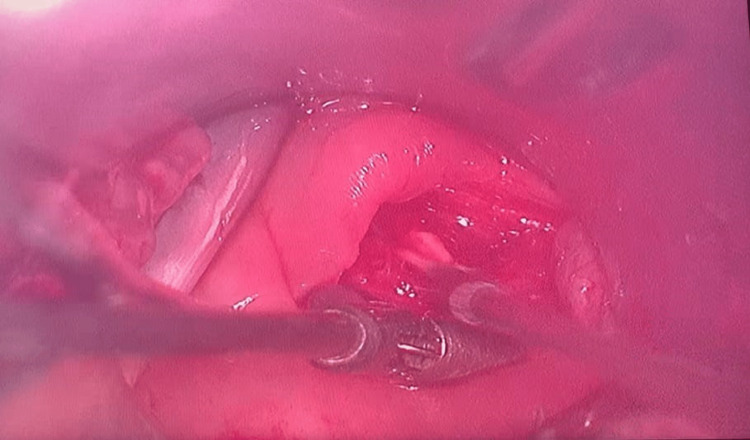
Transoral removal of the foreign body

**Figure 3:**
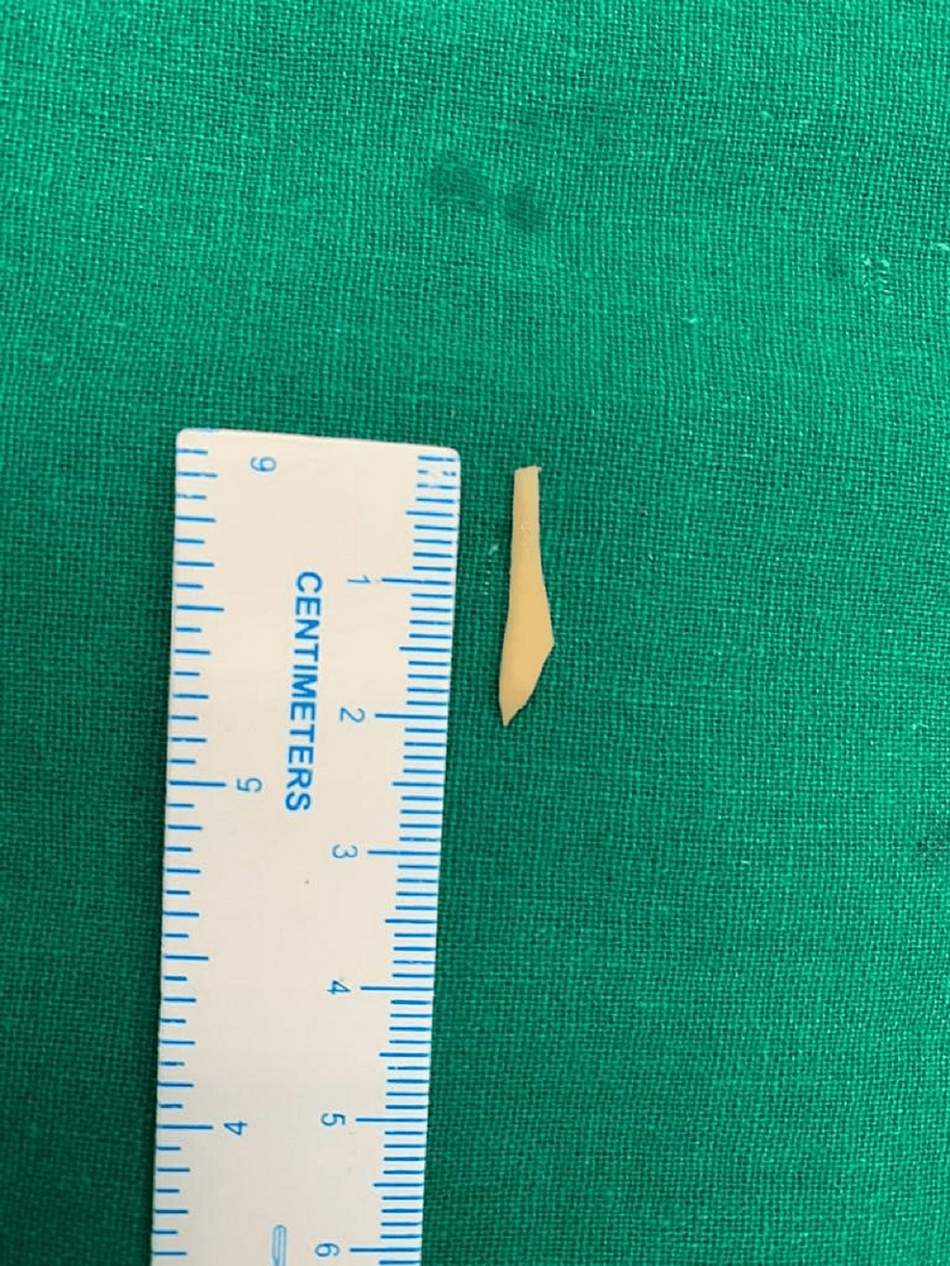
Sharp foreign body (chicken bone ) of 2.1 cm in length (removed)

A middle-aged person presented with a foreign body sensation in the throat and blood-tinged vomitus following a chicken meal two days ago. X-ray of the neck showed a foreign body at the level of hyoid bone with no widening of pre-vertebral soft tissue and no air-fluid level. Although an emergency check oesophagoscopy was done, the foreign body could not be retrieved due to oedematous oesophageal mucosa. The patient was given a course of antibiotics and steroids. CECT of the neck was done following this, which showed a 2.1 cm sharp foreign body seen extending obliquely and posteromedially from hyoid to apex of pyriform fossa, 1 cm lateral to the midline and 1 cm in depth from the posterior pharyngeal wall at C2 vertebral level, with no evidence of abscess or cellulitis, which showed that the foreign body has migrated to the retropharyngeal space. The patient was taken for a second look surgery via intra-oral approach, and a 0.5-cm oblique incision 1 cm from midline was made over the posterior pharyngeal wall. Foreign body chicken bone was retrieved from the retropharyngeal space. There was no evidence of perforating injury in the hypopharynx or oesophagus or any evidence of abscess, as shown in Figures 1-3.

Two children presented with similar complaints of fever, tachycardia, and difficulty in swallowing with restricted painful neck movements. One of them had a history of accidental foreign body ingestion two days back. On examination, there was a bulge in the posterior pharyngeal wall. X-ray of the neck and chest was done, which showed a radio-opaque foreign body with soft tissue swelling posterior to the pharynx with widening of pre-vertebral soft tissue pointing toward the retropharyngeal abscess. The blood picture showed leukocytosis. Incision and drainage were done via the intra-oral approach for one patient, and an open safety pin embedded in the posterior pharyngeal wall was retrieved. For the other child who presented with similar complaints but with no history of foreign body ingestion, X-ray findings seemed similar. For diagnosing further, a CT scan was done, which showed a hyper-dense foreign body 1 x 1 cm at the level of cricopharynx, and thickening of pre-vertebral soft tissue from the skull base to the superior mediastinum, with multiple air foci in the retropharyngeal space. The child underwent an emergency tracheostomy with incision and drainage (intra-oral approach). A metal piece present at the cricopharynx was removed via rigid oesophagoscopy. Postoperatively, the patient was started with the antibiotic cover. A feeding tube was inserted and decannulated after a week.

Another two cases, one in the paediatric age group and another in the geriatric age group with alcohol intoxication, presented with similar findings of tachycardia, fever, cough, retrosternal pain, and breathing difficulty following accidental ingestion of foreign body one day back. X-rays of the neck and chest were done for both patients, which showed radio-opaque foreign bodies in the right bronchus 0.5 cm and 1 cm from the hilum, with dense opacity in the right lung with prominent bronchovascular markings. Rigid bronchoscopy was done, and a stone piece and artificial denture were retrieved, respectively. Postoperatively, both patients were managed with oxygen support. X-ray was repeated after a three-day course of antibiotics, which showed normal findings.

Similar to the above two cases, another middle-aged lady presented with breathing difficulty, fever, and tachycardia, with a history of accidental ingestion of a pin, which she held between the teeth on attempting to drape the shawl. X-ray showed a radio-opaque foreign body located 2 cm from the hilum, with increased bronchovascular markings in the right lung. Rigid bronchoscopy was done, and the pin was retrieved. The patient was managed with oxygen support. Postoperative X-ray showed resolved changes.

An elderly person with some neuropsychiatric disorder presented with pain and difficulty in swallowing, retrosternal pain, and regurgitation of food. He also gave a history of loss of an artificial denture of his upper canine two days back. X-ray of the chest showed a radio-opaque foreign body at the oesophagus corresponding T4 vertebra. CT scan showed a hyperdense lesion in the mid-oesophagus with transmural thickening suggestive of oesophagitis. Although a check oesophagoscopy was done, which showed oesophageal mucositis, the foreign body could not be retrieved as its denture clip was adherent in the mid-oesophagus. Hence, we referred the case to a cardio-thoracic vascular surgeon, wherein the foreign was removed via thoracotomy.

## Discussion

In our study of 40 patients, 25 were male and 15 were female, with the paediatric age group having the highest prevalence, similar to a study conducted by Higo et al. [5]. All of them presented within the first 72 hours. Thirty out of 40 patients had similar symptoms, the most common being a sticky sensation in the throat for the oro-pharyngeal foreign body and breathing difficulty for the bronchial foreign body.

In our study showing atypical presentation, one patient had migration of foreign body (chicken bone) to retropharyngeal space, three developed aspiration pneumonia, and one patient presented with oesophagitis. This was similar to a study conducted by Hajiioannou et al., wherein there was an iatrogenic migration of an impacted pharyngeal foreign body of the hypopharynx to the pre-vertebral space [6]. Another study published by Qiu et al. showed similar migration of ingested sharp foreign body into the bronchus [7].

Hence the possibility of foreign body migration to deep neck space and lungs needs to be considered, as it leads to untoward complications if left untreated. Although diagnosis is based on history and symptoms, visualization of the size of the foreign body and its site, and dislodgement often require the use of radiological investigation. The anatomic location and imaging findings place a crucial role in guiding the surgeon. Anteroposterior and lateral radiographs of the neck and chest are quick and simple imaging methods preferred in the emergency department for the aerodigestive tract foreign bodies. It achieves a detection rate of 70-80% in assessing foreign bodies of the hypopharynx and cervical oesophagus. In the modern era, computed tomography scan (using soft tissues and bone window cuts) is the diagnosis of choice, as it detects even thin, small, minimally invasive, calcified foreign bodies. It is capable of even differentiating penetrating and migrating foreign bodies. CT scan also differentiates between cellulitis (infiltration in the subcutaneous tissue, subcutaneous fat septation) and abscess (rim-enhancing well-demarcated fluid collection). In our case series, two patients presented with parapharyngeal abscess and retropharyngeal abscess, and imaging findings played an important role in arriving at the diagnosis [8].

Literature shows that sharp foreign bodies will get horizontally aligned, and have a higher chance of penetrating the wall of the aerodigestive tract. Foreign bodies in the oropharynx are more likely to dislodge compared to foreign bodies in the laryngopharynx, probably due to the powerful movement of the tongue and also due to the larger diameter of the oropharynx [9]. The strong contraction of the hypopharyngeal and cricoesophageal muscles propels the food bolus into the oesophagus and this explains why higher rates of penetration occur in the hypopharynx and cervical oesophagus. Even our results were similar to the above studies [10]. Singh et al. found that the retention of foreign bodies for more than 24 hours was a major risk factor causing complications in children less than 10 years old, explaining the need for timely intervention [11].

A study conducted by Tashtush et al. showed an ingested sharp foreign body that presented as chronic oesophageal stricture and inflammatory mediastinal mass after 113 weeks [12]. This was similar to our study showing an atypical presentation of oesophagitis, with no prior history of foreign body ingestion. Hence, we recommend emergent intervention (preferably within two hours, but within six hours at the most) like a check oesophagoscopy and check bronchoscopy for these cases [13].

The method to remove gastrointestinal foreign bodies is associated with the type and size of the foreign body, position, time of confinement, and severity of symptoms [14,15]. Flexible endoscopy is the leading choice for the management of upper gastrointestinal foreign bodies, with a high success rate (greater than 94%) and fewer complications (0-6%) [16,17]. But rigid endoscopy is more appropriate to deal with sharp and long foreign bodies. The rigid endoscopy has some benefits for foreign bodies dislodged in the hypopharynx and upper oesophageal sphincter, while the lower oesophageal foreign bodies can be removed by flexible endoscopy [17].

## Conclusions

Sharp penetrating foreign bodies encountered in the aerodigestive tract pose a diagnostic challenge and demand surgeons' expertise. Relevant clinical history and atypical presentation of sharp penetrating foreign bodies should be addressed with the utmost caution, as foreign bodies migrate to deep neck space and bronchus and can result in untoward complications. Radiological investigations are often implicated for diagnosis, but in certain clinical scenarios where it cannot be ascertained, surgical exploration plays a pivotal role. Surgical challenges impose not only to identify the offending foreign body but also to deal with complications. Hence we need to be suspicious of the varied manifestation of the aerodigestive tract foreign bodies for early diagnosis and prompt treatment.
